# Advanced Fault Diagnosis and Health Monitoring Techniques for Complex Engineering Systems

**DOI:** 10.3390/s222410002

**Published:** 2022-12-19

**Authors:** Yongbo Li, Bing Li, Jinchen Ji, Hamed Kalhori

**Affiliations:** 1 School of Aeronautics, Northwestern Polytechnical University, Xi’an 710072, China; 2 School of Mechanical and Mechatronic Engineering, Faculty of Engineering and IT, University of Technology, Sydney, P.O. Box 123, Broadway, NSW 2007, Australia; 3 School of Mechanical and Mechatronic Engineering, The University of Technology Sydney, Sydney, NSW 2007, Australia

## 1. Introduction

Fault diagnosis and health condition monitoring have always been critical issues in the engineering research community. Over the past decade, with the rapid development of artificially structured materials, advanced sensing and data-driven intelligence algorithms, fascinating technical possibilities have been reported in the area of fault diagnosis and in the health condition monitoring of complex engineering systems. However, with the development of highly efficient intelligent algorithms, recent fault diagnosis and health monitoring strategies have become highly automated and are encountering sophisticated problems in terms of data availability, computational complexity, accuracy, etc. Meanwhile, combined with advanced intelligent algorithms, flourishing developments such as new sensing techniques, diagnostic approaches and the design of new types of metamaterials have also enabled significant advances and emerging opportunities in the field of system health condition monitoring. These studies will doubtlessly promote the reliability, availability and robustness of systems for the fault diagnosis and health monitoring of complex engineering systems.

This Special Issue of *Sensors* aims to collect research works encompassing the whole area of fault diagnosis and health monitoring techniques for engineering systems. This collection contains a total of 11 papers representing the current status of the research related to different methods of monitoring the health and reliability of engineering systems. 

Targeting the limitations of the original transition permutation entropy (TPE) method, Guo et al. [1] propose a multiscale transition permutation entropy (MTPE) method. Furthermore, considering the weaknesses of the proposed multiscale approach, the feature extraction ability of the MTPE method is further improved by proposing a composite multiscale transition permutation entropy (CMTPE) approach. Lastly, the researchers input the features extracted using the CMTPE method into an extreme learning machine (ELM) to perform the fault diagnosis of a bearing. 

Bykerk et al. [2] used vibro-acoustic sensors for detecting leaks in the water distribution mains of an urban area. The real-time data collected from the extensive deployment of the vibro-acoustic sensors across a sprawling metropolitan city were used to monitor the presence and absence of pipe leaks using a convolutional neural network (CNN) after pre-processing via short-time Fourier transform (STFT). Different external factors, such as pipeline size, pipeline material and the soil condition around the pipe, are also taken into consideration. 

Asadi et al. [3] designed a Takagi–Sugeno (TS) fuzzy-based sliding mode observer (SMO) to reconstruct the faults in actuators and sensors installed in a nonlinear system subjected to unknown external disturbance. A non-quadratic Lyapunov function (NQLF) and fmincon function were used to guarantee the stability of the proposed SMO as a matlab optimization tool. The influence of unknown disturbances and uncertainties are minimized by utilizing a performance criterion. The proposed method provides better accuracy, less conservative optimization conditions and improved generality in comparison to other existing state-of-the-art methods. 

Hu et al. [4] combined piecewise aggregate approximation (PAA) with complete ensemble empirical mode decomposition (CEEMDAN) to alleviate the high memory requirements and low computational efficiency of the CEEMDAN method in bearing fault diagnosis. Vibration signals were used to study the efficacy of the proposed method. An enhanced bearing fault diagnosis performance was obtained using the proposed method. 

Aiming to solve the problem of unavailable data in online fault detection in rolling element bearings, a multiscale deep support vector data description (Deep-SVDD) approach is proposed by Kou et al. [5] By utilizing data enhancement technology, training data were transformed into multiple subspaces. Then, a subsequent clustering algorithm was utilized to enhance the robustness of the features. Lastly, the proposed Deep-SVDD model was constructed to achieve the online monitoring of the health of rolling element bearings. The proposed method can be utilized to detect incipient faults in a bearing. 

A new oversampling algorithm, namely, MeanRadius-SMOTE, is proposed by Duan et al. [6] for diagnosing mechanical faults regarding unbalanced data. The newly proposed method can effectively avoid the generation of useless and noisy samples and solve the multiclassification problem regarding different mechanical faults. A complete diagnosis of the faults in mechanical equipment can be achieved using the proposed method. 

Mao et al. [7] addressed the challenges of an incomplete training dataset using a cross-domain intelligent fault diagnosis approach and proposed a novel deep learning approach called the partial transfer ensemble learning framework (PT-ELF). After substituting the missing health states with another dataset, the proposed method was able to address the variable data distribution challenge by training a weak global classifier and two partial domain adaption classifiers. Lastly, a specific ensemble strategy was used to combine these classifiers for fault diagnosis. 

Aldawood et al. [8] developed a self-vibration-powered energy harvester sensor system to tackle the environmental threat posed by unused batteries in battery-powered sensors in wireless sensor networks (WSN). Dual moving magnets bordered by coil windings were used for power and signal generation in a harvester sensor unit. A radio frequency (RF) transmitter is operated using the power generated from the harvester, and the generated signal from this sensor is transmitted as the vibration signal. Lastly, a custom-made APP is utilized to detect faults in this system. 

A 1D dilated convolutional neural network (1-DDCNN) is proposed by Chen et al. [9] for the fault diagnosis in an aircraft retraction/extension (R/E) system.Aiming to solve the limited feature information extraction and fault diagnosis ability of 1-DCNNs, multiple feature parameters have been used. Moreover, the main fault mode of the R/E system for aircraft landing gears has been studied, specifically exploring its working principal and the influence of convolutional kernel size on the classification accuracy. 

Lee et al. [10] studied the optimal sensor selection criteria in a multi-sensor-based fault diagnosis of a roll-to-roll printed electronics system. Data are collected for four major defects of a Gravure roll-to-roll printed electronic system with three triaxial acceleration signals. Smart data were formed from the collected raw data obtained by a sensor data efficiency evaluation; a sensitivity evaluation for axis selection considering the directional nature of faults; and feature variable optimization using the feature combination matrix method. The progressive application of the aforementioned phases enhanced the fault diagnosis results in terms of accuracy, calculation time, predictive ability and data storage capacity.

Pan et al. [11] proposed a new method to investigate the mitigation of commonly occurring rotor–stator rub impact faults in aero-engines. A pre-strained, two-way shape memory alloy (SMA) wire was used in the design of a current-driven active control actuator to mitigate the occurrence of rub impact faults. The feasibility of the proposed scheme is verified by different properties of the used NiTi wires. Finally, a prototype of the schemed actuator was designed and manufactured for testing under various conditions. The status of the rub impact fault was monitored using an acoustic emission sensor.

On behalf of all the editors of this Special Issue, we would like to extend our heartiest gratitude for the contributions from the authors to this project. We would also like to extend our sincere thanks to all the reviewers and members of the editorial board of *Sensors*. 
